# Speech perception in noise: Impact of directional microphones in users of combined electric-acoustic stimulation

**DOI:** 10.1371/journal.pone.0213251

**Published:** 2019-03-06

**Authors:** Tobias Weissgerber, Timo Stöver, Uwe Baumann

**Affiliations:** 1 Audiological Acoustics, ENT Department, University Hospital Frankfurt, Frankfurt am Main, Germany; 2 ENT Department, University Hospital Frankfurt, Frankfurt am Main, Germany; University of Leeds, UNITED KINGDOM

## Abstract

**Objectives:**

Combined electric-acoustic stimulation (EAS) is a well-accepted therapeutic treatment for cochlear implant (CI) users with residual hearing in the low frequencies but severe to profound hearing loss in the high frequencies. The recently introduced SONNETeas audio processor offers different microphone directionality (MD) settings and wind noise reduction (WNR) as front-end processing. The aim of this study was to compare speech perception in quiet and noise between two EAS audio processors DUET 2 and SONNETeas, to assess the impact of MD and WNR on speech perception in EAS users in the absence of wind. Furthermore, subjective rating of hearing performance was registered.

**Method:**

Speech perception and subjective rating with SONNETeas or DUET 2 audio processor were assessed in 10 experienced EAS users. Speech perception was measured in quiet and in a diffuse noise setup (MSNF). The SONNETeas processor was tested with three MD settings omnidirectional/natural/adaptive and with different intensities of WNR. Subjective rating of auditory benefit and sound quality was rated using two questionnaires.

**Results:**

There was no significant difference between DUET 2 and SONNETeas processor using the omnidirectional microphone in quiet and in noise. There was a significant improvement in SRT with MD settings natural (2.2 dB) and adaptive (3.6 dB). No detrimental effect of the WNR algorithm on speech perception was found in the absence of wind. Sound quality was rated as “moderate” for both audio processors.

**Conclusions:**

The different MD settings of the SONNETeas can provide EAS users with better speech perception compared to an omnidirectional microphone. Concerning speech perception in quiet and quality of life, the performance of the DUET 2 and SONNETeas audio processors was comparable.

## Introduction

Combined electric-acoustic stimulation (EAS) is a well-accepted therapeutic treatment for cochlear implant (CI) users with residual hearing in the low frequencies but severe to profound hearing loss in the high frequencies, i.e. ski slope-type hearing loss [1]. The unilateral combination of electric stimulation of the high frequencies via a CI and acoustic stimulation of the low frequencies via a hearing aid enables users to achieve better speech perception in quiet [2,3], in noise [2–7], and better sound localization [7,8] than does electric-only stimulation.

However, speech perception in noise is still a challenge for many hearing implant users. Beside developments which are focused on sound coding strategies and electrode design, considerable improvements may also be reached by using front-end processing. Using directional microphones is a well-established concept of front-end processing in hearing aids, which has been successfully incorporated since the 1990s [9,10]. The first adaptive beamforming algorithm integrated into CI audio processors was commercially introduced in 2005 [11]. In studies assessing the performance of this adaptive beamformer for speech perception with multiple noise sources in a laboratory environment, improvements in the signal-to-noise ratio (SNR) were large compared to a mild fixed directionality to the front (sub-cardioid), ranging from 3.9 to 6.5 dB, depending on the specifications of the test setup used: Spriet et al. 2007 ([11], 6.5 dB in speech-weighted noise); Brockmeyer and Potts 2011 ([12], 4.2 dB), Gifford and Revit 2010 ([13], 3.9 dB), Hersbach et al. 2012 ([14], 5.3 dB). Moreover, recent studies reported that directional microphone technology significantly improves speech reception thresholds (SRTs) in CI users ([15–18]).

The MEDEL (Innsbruck, Austria) DUET 2 processor [19] was the second generation of a CI combined with hearing aid amplification/acoustic driver introduced in 2009. With the successor SONNETeas in 2015, a complete redesign of the acoustic processing was carried out, which provided more amplification and less distortion. In addition, the fitting software was completely overworked and provided new features. This audio processor also offers different microphone directionalities (MD) and wind noise reduction (WNR) as front-end processing. It is well known that beamformers in general augment wind noise [20,21]. Therefore, the activation of WNR is oftentimes desirable and recommended by the manufacturer.

Since WNR is provided by decreasing gain in the lower frequencies, especially for EAS users this strategy might influence performance. Users of EAS are a special population which rely heavily on low frequency acoustic information for the enhancement of speech perception in noise [22]. Therefore, reduction of low frequency information may lead to performance being negatively impacted. In addition, directional microphone settings inherently decrease gain in the low frequency region and equalization is necessary to compensate this potential detrimental effect. The manufacturer of hearing aid or cochlear implant devices must balance carefully these two components of the acoustic preprocessing against each other. Therefore, the impact of MD and WNR on speech perception in EAS users is of special interest. However, this issue was not addressed comprehensively so far in previous studies.

Consequently, the aim of this study was to compare speech perception in quiet and in noise in EAS subjects using different settings of front-end processing. Furthermore, speech perception was compared with the predecessor of the audio processor [19,23] and subjective rating of auditory benefit with either device was assessed using questionnaires.

Three hypotheses were addressed in the manuscript. First, it was hypothesized that speech perception with DUET 2 and SONNETeas MD omnidirectional are comparable. Second, there is a significant impact of MD for speech perception in noise using the SONNETeas processor. And third, there is no impact of WNR on speech perception in the absence of wind noise.

## Materials and methods

### Subjects

**Fig 1:**
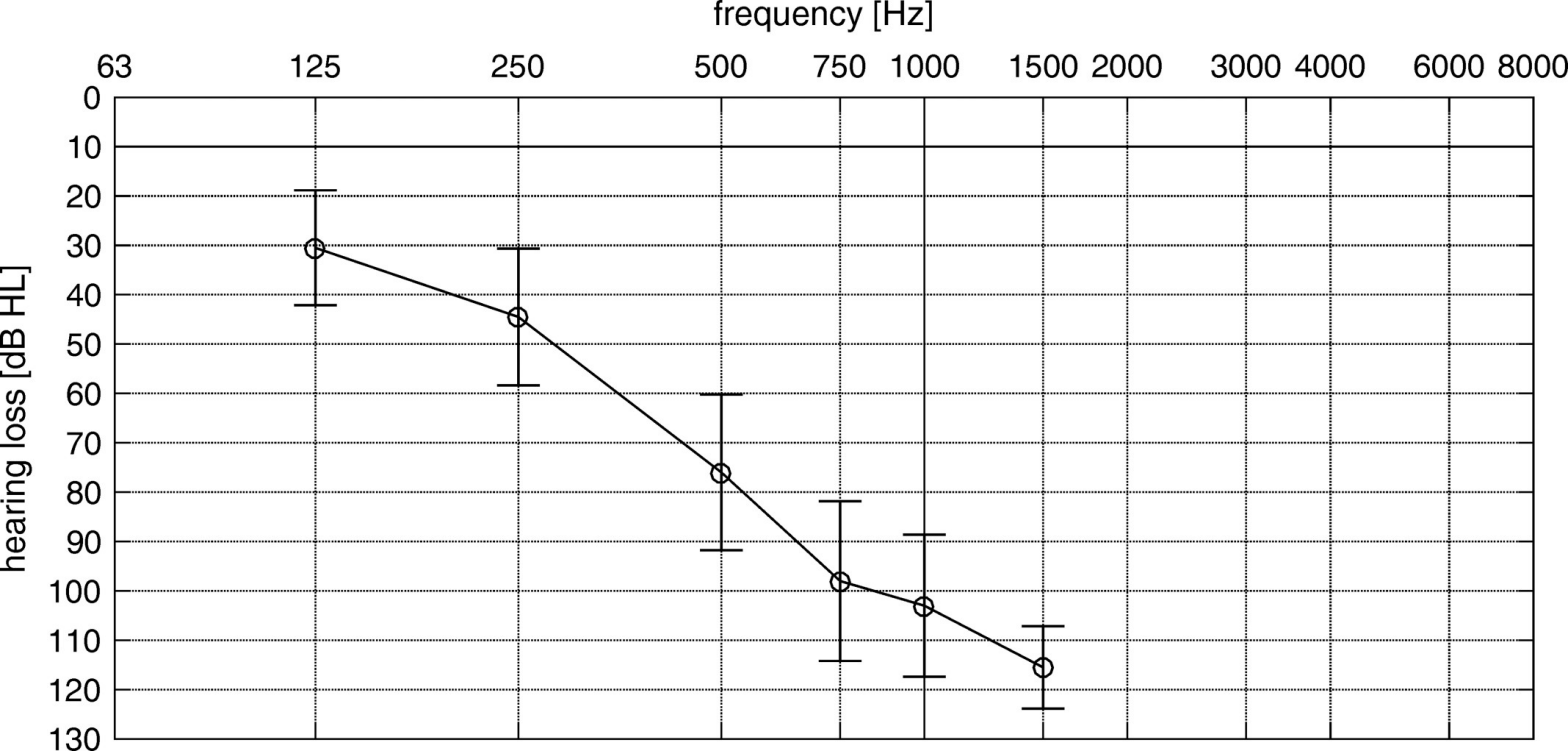
Pure-tone thresholds (errorbars with mean and standard deviation) of the ipsilateral ear. None of the subjects showed any residual hearing above 1.5 kHz.

Ten subjects took part in the study. Their mean age at time of implantation was 51.3 years (37–67 years); their mean age at time of testing was 55.1 years (43–68 years). Seven subjects were implanted unilaterally and 3 were implanted bilaterally (i.e. EAS in both ears). All subjects were experienced users (>12 months) of the DUET 2 audio processor. Eight were tested in their right ear and 2 were tested in the left ear. Three subjects had all 12 electrode contacts switched on, four had 11 contacts switched on, two had 10 contacts switched on, and one had 9 contacts switched on. All subjects were either implanted with a PULSAR, SONATA, or CONCERTO implant. Mean unaided pure-tone thresholds in the implanted ear are shown in Fig 1. Pure-tone thresholds were assessed at the first study appointment. Thresholds for frequencies with no response were set to 120 dB HL. To ensure the feasibility of the speech test in noise, all subjects had a score of at least 40% on the Freiburg Monosyllabic test in quiet (EAS ear only, contralateral ear blocked) and fluency in German language. In all test conditions, only the ipsilateral ear using EAS was tested. In bilateral EAS subjects, the ear with better monosyllable score was chosen as ipsilateral. The contralateral ear was double-blocked with earplugs and closed circumaural headphones (Sennheiser HDA200). Subjects received an allowance for the participation in the study.

### CI audio processor settings

The DUET 2 audio processor has a fixed omnidirectional microphone directionality whereas the SONNETeas processor offers three modes of microphone directionality (MD):

- Omnidirectional mode: only the front microphone is active; the rear microphone is disabled.
- Natural mode: the signals from the front and the rear microphone are combined to form a microphone with a fixed directional characteristic to the front (frequency-dependent, mimicking the pinna). The natural MD is implemented as a simple delay-and-sum MD (first-order differential array).
- Adaptive mode: the signals from the front and the rear microphone are combined to adjust the direction of minimal sensitivity (i.e. highest suppression) adaptively to the direction of a noise source. This works only if the noise source is behind the listener. The attenuation of noise is frequency and location selective.

Polar plots of the MD modes “natural” and “adaptive” are given in [24]. To overcome the drawback of higher sensitivity for wind noise in directional microphones compared with omnidirectional microphones, the SONNETeas features a WNR algorithm. The audio signals of front and rear microphones are analyzed and filtered when wind noise is detected. The SONNETeas WNR function can be operated in any of the 3 following modes: off, mild, or strong. According to the selected mode, the threshold of the wind noise detector is different (higher for mild, lower for strong). The higher the threshold, the more wind power is needed for wind detection. The WNR algorithm applies 2 different mechanisms: the first mechanism combines the two low-pass signals of the microphones in a way to achieve a basic WNR. The spectral shape of the audio signal is not changed. The second mechanism attenuates the low-pass area and, thus, alters the spectral shape of the audio signal (i.e. low-frequency attenuation). The suppression network has a fade-in/fade-out procedure for switching smoothly between the WNR mode and the bypass mode. If WNR is enabled but no wind is detected, the microphone signal will not be modified.

### Speech tests

In all test conditions, only the ipsilateral ear using EAS was tested. The contralateral ear was double-blocked with earplugs and closed circumaural headphones (Sennheiser HDA200).

Speech perception in quiet was assessed with Freiburg Monosyllables test (FMS, [25]). The FMS score in quiet was measured in free-field conditions at a sound pressure level of 65 dB SPL. Speech was presented from front at a distance of 1m to the test subject. Each subject performed one list (list number 1) for training before the start of the actual testing. For each test condition, two test lists with 20 monosyllables each were conducted and the number of correct words was summed up and divided by 40 to achieve the word recognition score in percent. The order of test lists was randomized. Since the cohort of subjects consisted of high performers (FMS scores between 60% and 95% with DUET 2 processor), speech perception ceiling effects in quiet with the FMS test were expected. Therefore, the FMS test served mainly as reference and additional verification of proper fitting of the SONNETeas processor.

Speech perception in noise in free-field conditions was assessed with Oldenburg Sentence Test in Multi-Source Noise Field (OLSA MSNF) condition [6,26]. Speech signal was presented from front (0°) with a distance of 1.75 m to the listening position. Four adjacent speakers were used for the presentation of the speech signal. The MSNF with four virtual sound sources was realized by means of wave field synthesis [27]. Four noise sources were created at approx. ±30° and ±150° in a distance of 1.25 m to the middle of the listener’s head. Signal for the four noise sources was continuous Olnoise. The Olnoise signal was generated by the summation and averaging of 30 randomly time-wise shifted OLSA test sentences. Therefore, OL-noise showed only very weak temporal modulation. The methods of summation and averaging kept the short-term spectrum equal to the OLSA sentences. The four noise sources were temporally decorrelated by shifting the starting point of each of the four noise channels by two seconds to create a pseudo-diffuse noise field. The sound pressure level of the noise signal was fixed at 65 dB SPL and speech level was set adaptively according to the number of words perceived correctly to measure the speech reception threshold (SRT). The adaptive procedure proposed by Brand and Kollmeier [28] was used. The initial SNR was +5 dB. The OLSA was conducted in the closed-set mode, the subject had to mark the words on a touch screen monitor. No answer was mandatory. If the subject is uncertain, it is allowed to guess or to proceed further by touching the “ok” button. Each subject performed one list (list number 1) for training before the start of the actual testing. For each test condition, one test list with 30 sentences was conducted. The order of test lists was randomized. Further details and normative data for the setup are given in [26].

### Questionnaires

The HISQUI_19_ [29] is a questionnaire for CI users to quantify the auditory benefit they derive from using their device in everyday listening situations via perceived sound quality. It contains 19 items of equal weight which are answered on a 7-point Likert scale (“Always” to “Never”). Qualifying the level of benefit is done by adding up the scores: a total score of less than 30 indicates very poor sound quality, 30–59 is poor sound quality, 60–89 is moderate sound quality, 90–109 is good sound quality, and 110–133 is very good sound quality. Auditory benefit improves with increasing sound quality score.

The SONNETeas questionnaire is a custom questionnaire specifically designed for this study to collect subjective feedback from SONNETeas users about which MD and WNR settings they preferred in different listening situations and why they preferred them. It includes questions about positive and negative features of the SONNETeas, program-changing habits, and about subjectively perceived sound quality. The questionnaire consisted of three parts: 1) four open-ended questions (general satisfaction, suggestions for modifications of the processor, sound quality, preference of program), 2) ten statements to which subjects are asked to provide their degree of agreement/disagreement with on a 5-point Likert scale (“I totally agree” to “I totally disagree”), 3) the yes/no question: “Would you recommend EAS to other potential candidates?”. All subjects were informed about the handling of both questionnaires and potential misunderstanding of items was cleared prior filling out.

### Procedure and intervals

Testing was done at two intervals. The second interval was 14–28 days after the first interval. At the first interval, the functionality and the fitting of their own DUET 2 speech processor was assessed. Afterwards, subjects completed the following tasks while using their own DUET 2:

- Speech perception in quiet (Freiburg Monosyllables, FMS, [25])
- Speech perception in noise (Oldenburg Sentence Test in Multi-Source Noise Field, OLSA MSNF, [6,26])
- Auditory benefit (Hearing Implant Sound Quality Index 19, HISQUI_19_, [29]).

After completing the tests and questionnaire, subjects left their DUET 2 processors at the clinic for the duration of the study and received a SONNETeas processor, which was fitted with the following three settings:

- MD natural / WNR mild (default setting as recommended by the manufacturer)
- MD omnidirectional / WNR off
- MD always adaptive / WNR mild.

All subjects were informed about the handling of the SONNETeas test processor and differences to their DUET 2 processor (e.g. battery change, ear hook replacement, on/off switching, lighting signals). The position of the different processor settings on the remote control (FINETUNER) was explained and demonstrated. For the time between intervals, subjects were instructed to try all three settings in different everyday situations to discover which settings they preferred using in which listening situations. Especially in situations with wind, program switching between WNR off and WNR mild was encouraged.

At the second interval, subjects used SONNETeas to complete all of the same tests they had completed in the first interval with the DUET 2 and, additionally, complete a SONNETeas questionnaire (see Table 1) about the subjective performance using this device. FMS were tested in omnidirectional condition with the 3 settings WNR off/mild/strong. To assess the effect of MD on speech perception in noise, OLSA MSNF was tested in all available MD settings with mild WNR. To assess the effect of WNR on speech perception in noise, OLSA MSNF with MD omnidirectional was tested in all three WNR settings. No wind was present in the tested conditions in quiet and noise. The different WNR settings were tested to verify that there is no impact of WNR on speech perception in the absence of wind noise (hypothesis 3).

**Table 1:** SONNETeas questionnaire (number of answers per category per question).

*Question*	*CA*	*MA*	*NAD*	*RD*	*CD*	*?*
The processor is comfortable to wear in everyday life.	6	1	1	1	0	1
I like the appearance of the SONNETeas.	4	5	1	0	0	0
The SONNETeas is easy to handle.	4	3	2	1	0	0
The SONNETeas offers me a new and better listening experience.	3	5	1	1	0	0
Microphone directionality reduces the listening effort in noisy environments.	4	3	2	1	0	0
The wind noise reduction system reduces the listening effort in windy environments.	2	5	2	1	0	0
I prefer to use the SONNETeas without microphone directionality and wind noise reduction.	0	4	3	1	2	0
The SONNET sometimes produces distracting noise.	0	1	2	3	4	0
I perceive the sound quality of the SONNETeas as very natural.	4	4	1	1	0	0
Overall, I am very satisfied with the SONNETeas.	3	5	1	1	0	0

CA = completely agree, MA = mostly agree, NAD = neither agree nor disagree, RD = rather disagree, CD = completely disagree, ? = data missing

### Ethics and consent

The study was conducted according the Declaration of Helsinki and was approved by the local institutional review board (University Hospital Frankfurt/Main, reference number 246/15). All subjects gave their written informed consent before the start of any study-specific procedure.

### Statistics

Descriptive statistics using mean and standard deviation were used to report subject demographics (e.g. age and gender), and to present test results of the speech tests and questionnaires. Normality was checked for each individual test variable using Shapiro-Wilk test. The hypothesis of normality was not rejected for all test variables in both statistical tests. Therefore, parametric tests were used for statistical analysis.

For more than two paired comparisons, the overall effect was confirmed by a repeated-measures analysis of variances (RM-ANOVA). For multiple comparisons (post-hoc tests or multiple paired *t*-tests), *p*-values were adjusted with the Bonferroni-Holm correction method. A *p*-value < 0.050 was considered significant. IBM SPSS Statistics 22 (IBM, Armonik, New York) was used for the analyses.

## Results

### Speech perception in quiet

**Fig 2:**
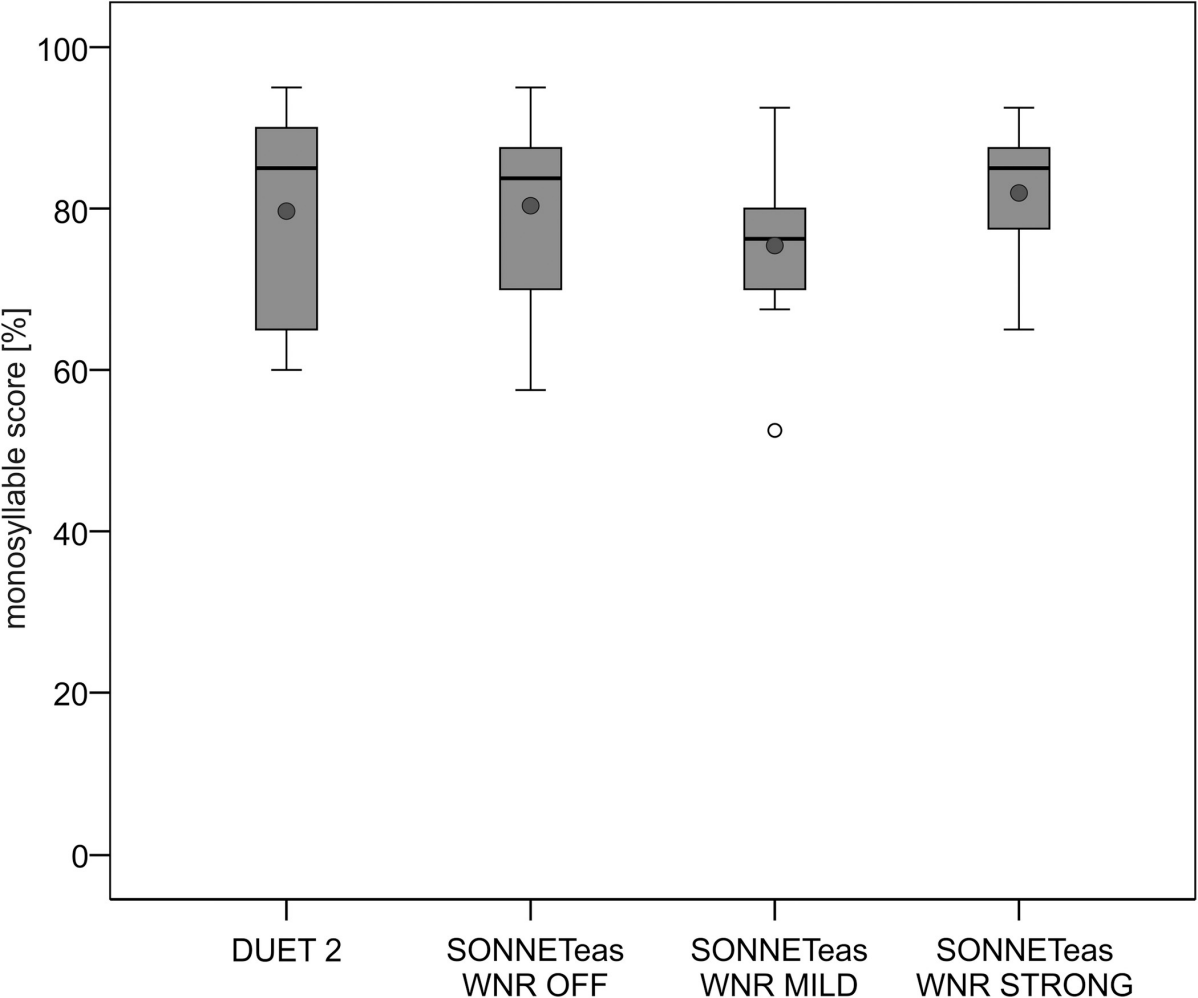
Boxplots of monosyllable scores obtained with the DUET 2 audio processor and the SONNETeas processor in three different WNR settings. MD setting was omnidirectional. Grey circles indicate the mean value, open circles indicate outliers.

The results of speech perception in quiet are shown in Fig 2. Mean FMS score ranged between 75.8±10.7% (Sonnet with mild WNR) and 80±12.8% (DUET 2). There was no significant effect of audio processor (*t* = -0.225, df = 9, *p* = 0.827). A repeated-measures ANOVA showed a significant effect of WNR on speech perception in quiet (F = 5.034, df = 2, *p* = 0.018). This effect was so small that in post-hoc tests using adjusted *p*-values for multiple comparisons (Bonferroni-Holm correction) no significant difference between WNR conditions was confirmed (WNR off vs. mild: *p* = 0.090; WNR off vs. strong: *p* = 0.394. WNR mild vs. strong: *p* = 0.090).

### Speech perception in noise

**Fig 3:**
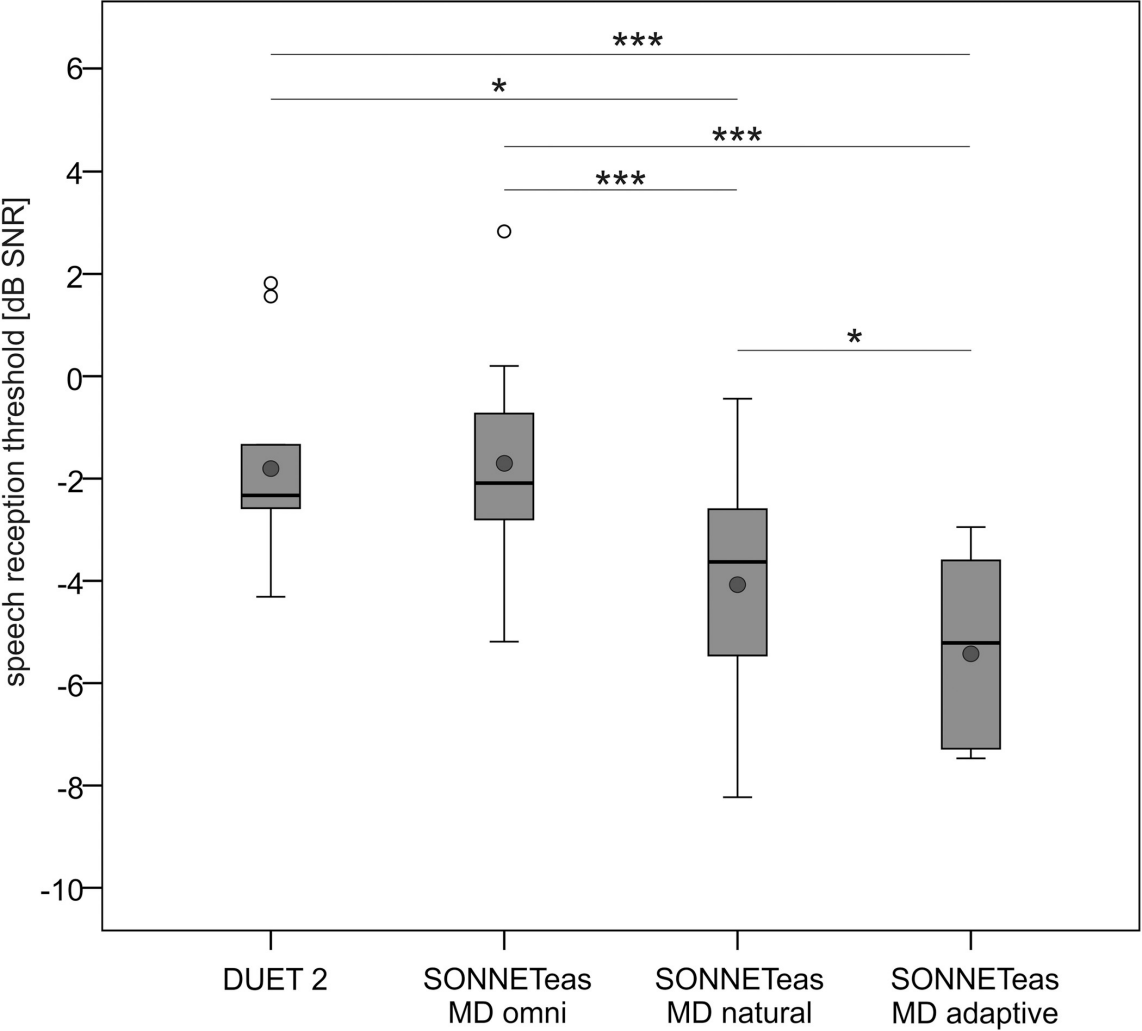
Boxplots of speech reception thresholds with audio processors DUET2 and SONNETeas with MD omnidirectional, natural and adaptive directional microphone. WNR setting of SONNETeas was always mild (i.e. default setting). Grey circles indicate the mean value, open circles indicate outliers. Significant differences between conditions are indicated with asterisks. ***: *p* < .001; **: *p* < .005; *: *p* < .050.

The SRT results obtained in MSNF with the DUET 2 processor and with the SONNETeas processor in all MD settings with WNR setting mild are shown in Fig 3.

#### Comparison of audio processors

SRT with the DUET 2 was -1.7±2 dB SNR and with the SONNETeas using omnidirectional microphone and WNR off was -2.3±1.9 dB SNR. There was no significant difference between audio processors (*t* = 0.742, df = 9, *p* = 0.477). SRTs with fixed MD natural (SONNETeas default setting) were 2.2 dB (*t* = 3.146, df = 9, *p* = 0.024) better and with adaptive MD 3.5 dB (*t* = 4.935, df = 9, *p* = 0.003) better compared with the DUET 2 processor.

#### Impact of microphone directionality

Mean SRTs with SONNETeas processor with WNR mild were -1.7±2.1 dB SNR (MD omnidirectional), -3.9±2.2 dB SNR (MD natural) and -5.3±1.8 dB SNR (MD adaptive). There was a significant effect of microphone directionality (F = 34.254, df = 2, *p* < 0.001). Post-hoc tests with Bonferroni-Holm correction showed that mean SRT with MD natural was significantly better than with MD omnidirectional (*p* < 0.001), mean SRT with MD adaptive was significantly better than with MD omnidirectional (*p* < 0.001) and MD natural (*p* = 0.026).

#### Impact of WNR

**Fig 4:**
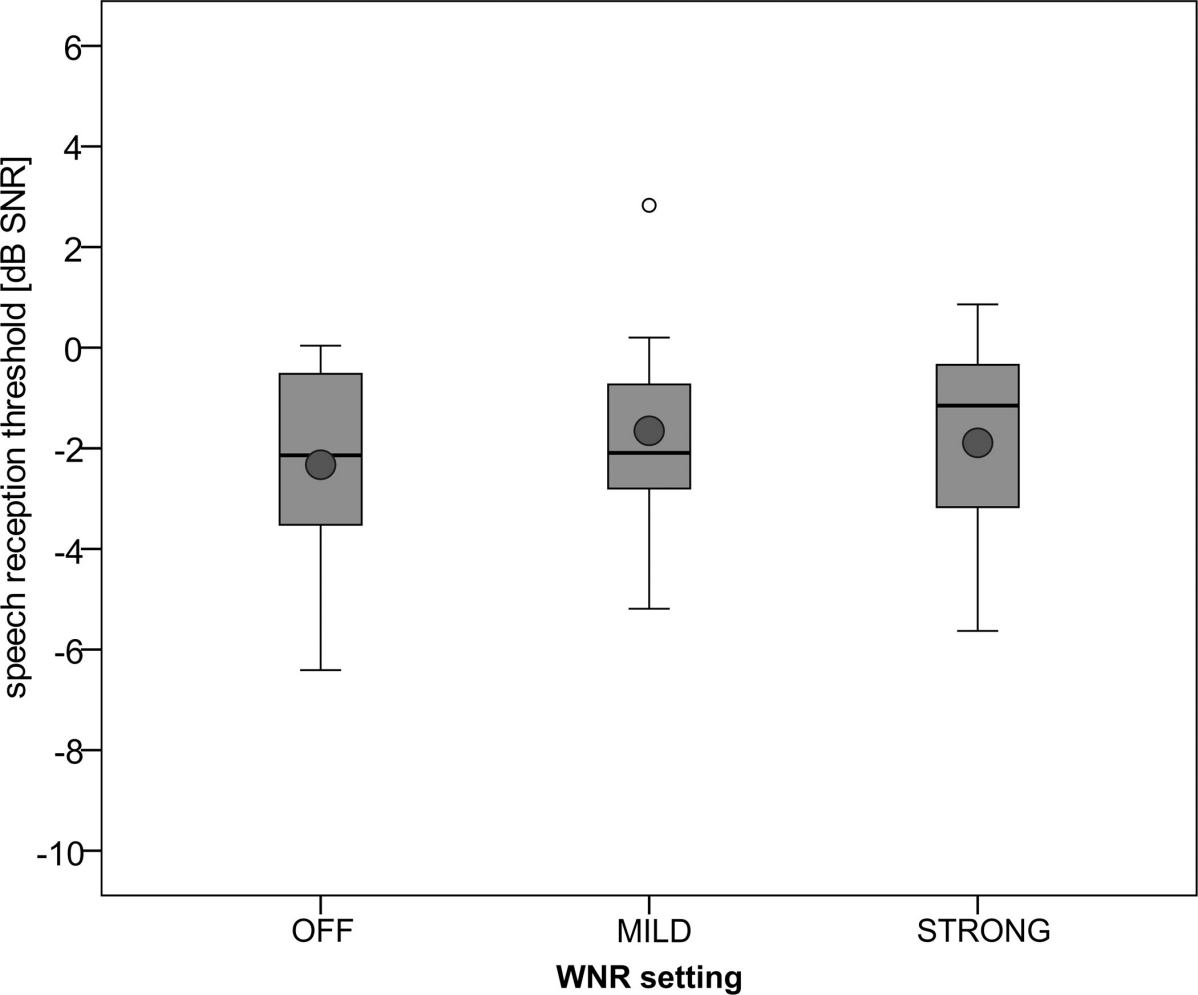
Boxplots of speech reception thresholds with audio processor SONNETeas with WNR off/mild/strong. MD was always omnidirectional.

Boxplots with SRTs obtained in MSNF with the SONNETeas processor in all MD settings with WNR settings off/mild/strong (MD omnidirectional) are shown in Fig 4. Mean SRTs were -2.3±1.9/-1.7±2.1/-1.9±2.1 dB SNR. In the present study cohort we could not observe a detrimental effect of WNR on speech perception (F = 1.031, df = 2, *p* = 0.377).

### Questionnaires

**Fig 5:**
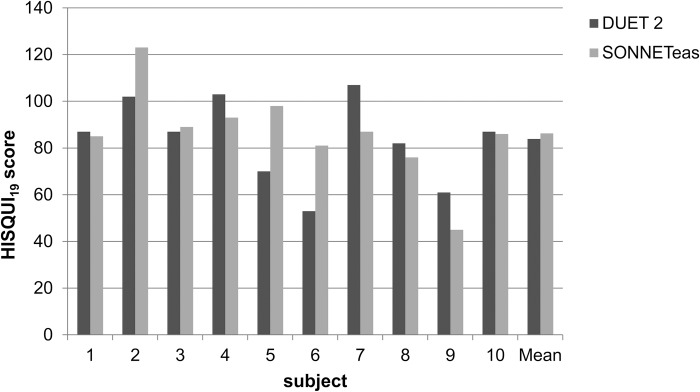
Mean HISQUI19 scores by device (DUET 2 and SONNETeas) by subject.

The results of the HISQUI_19_ questionnaire are shown in Fig 5. In the HISQUI_19_ questionnaire, subjects had a mean “moderate” self-perceived auditory benefit with both the DUET 2 (mean: 83.9±18.0) and the SONNETeas (mean: 87.2±19.6) processor. No significant effect of audio processor was found (*t* = -0.595, df = 9, *p* = 0.566).

In the SONNETeas questionnaire, subjects gave feedback regarding the comfort, appearance, ease of handling, and function of the SONNETeas. The results of the questionnaire are shown in Table 1. Of the three settings which subjects used between intervals, subjects preferred MD/WNR settings natural/mild and omni/off for everyday use. Nine out of ten subjects would recommend EAS treatment to a potential candidate “if adequate medical guidance is given”; one out of ten would definitely recommend it.

## Discussion

This study investigated the performance of the SONNETeas audio processor compared with the performance of the predecessor device (DUET 2) in experienced EAS users. Speech perception was tested in quiet and in noise with MSNF and subjective feedback about the two processors was collected.

### Effect of audio processor

One objective of the study was to assess whether the SONNETeas performed the same or better as the DUET 2 in speech tests in quiet and in noise. The DUET 2 is based on omnidirectional microphone technology only. Speech scores in quiet were comparable between both audio processors. In noise, there was also no significant difference between the audio processors with omnidirectional MD setting.

In addition to the integration of directional microphones, the SONNETeas device also introduced a more advanced setting of the amplification of the acoustic signal with the possibility of individual adjustment in 6 frequency bands ranging from 125 Hz to 1.5 kHz. Furthermore, the quality of the amplifier/transducer improved in terms of distortion (third harmonic distortion), maximum gain and noise floor. Therefore, a more precise transfer of the signal information contained in the acoustical pathway was assumed.

Using the SONNETeas processor with default MD/WNR setting natural/mild, SRTs were better for the SONNETeas condition.

### Effect of front-end processing

Using the SONNETeas processor with default MD/WNR setting natural/mild, SRTs in noise were better than in omnidirectional condition. Using the adaptive MD setting the speech perception was superior to all other test conditions, even in the diffuse MSNF setup. No effect of WNR on FMS in quiet or on SRTs in noise was found in the absence of wind. The reason for this is presumably that the WNR algorithm is working as intended by the manufacturer so that (according to hypothesis 3) WNR remains bypassed in all settings (off/mild/strong) as long as no wind is present.

The results indicate that the front-end feature MD of the SONNETeas is also suitable for EAS users, and that they can obtain significant benefit from these features. However, it is already known that the benefit using directional microphones has some limitations. The impact of beamforming algorithms in everyday life situations highly depends on the listening situation (i.e. free-field or reverberation, number and location of noise sources, signal-to-noise ratio, etc.). Directional microphones are most effective in cases when the CI user is facing the speech target and noise sources are behind the listener. On the other hand, CI users sometimes complain about the “encapsulation” caused by directional microphones, since acoustic information which is present in the real world is not delivered to the ear of the implant user. This could explain the result of the present study that the CI users either preferred MD natural or omnidirectional in everyday life.

### Comparison of the results with previous studies

The presented study is the first study which investigated the effect of directional microphones on speech perception in EAS users. Previous studies were oftentimes focused on the impact of directional microphones on speech perception in noise in users of electric stimulation (e.g. [11,15–18,26]). In MSNF condition using exactly the same test paradigm, normal hearing subjects had a mean SRT of -10 dB SNR and bilateral CI users with a moderate directional microphone to the front (sub-cardioid, Cochlear CP810 audio processor) had a mean SRT of -4.1 dB SNR [26]. In the present study, unilateral EAS users showed a mean SRT of -3.6 dB SNR (MD natural) and, thus, were with one ear almost as good as users of electric stimulation in two ears. This result in EAS subjects is potentially related to the additional access to low frequency information provided by the acoustic component and thus collaboration of fundamental frequency contour cues with the slight enhancement of the frontal sound source delivered with MD natural.

Wimmer et al. [16] compared SRTs in noise between SONNET and OPUS 2 (predecessor of the SONNET for electric-only stimulation) audio processors. They report an SRT improvement using SONNET with MD natural of 3.6 dB in a test condition with a single noise source at 180°. Honeder et al. [18] found an SRT improvement of 4.3 dB (MD natural) and 6.1 dB (MD adaptive) compared with MD omnidirectional in a setup with two noise sources at ±135°. Both studies tested unilateral CI users without acoustic stimulation.

In a previous study the authors assessed the impact of directional microphones (Cochlear CP810 audio processor) on speech perception in a moving noise condition [17]. In this study, an SRT improvement of up to 8 dB was observed. However, the presence of only one noise source in a free-field condition is a rather seldom situation in everyday life. Therefore, in the present study a more diffuse noise condition was used, where two of the four noise sources were in the frontal hemisphere of the test subject. Consequently, the beneficial effect of directional microphones was less pronounced than in other studies. The measured benefit also depends on the baseline condition. Weissgerber et al. 2017 [26] found even less beneficial effects of microphone condition in diffuse noise using the Cochlear CP810 audio processor. In this study, the baseline condition was a fixed directional microphone (sub-cardioid) whereas in the present study the baseline was an omnidirectional microphone.

### Auditory benefit and sound quality

Concerning the subjective auditory benefit as evaluated by the HISQUI_19,_ six users performed the same (i.e. a mean HISQUI score difference of less than 5 points) or better with the SONNETeas. This matches subjects’ impressions of the device–with some saying it was too short a time to fully get used to the SONNETeas–perhaps with more time this preference might change significantly towards the SONNETeas.

## Conclusions

The front-end features of the SONNETeas can provide experienced EAS users with significantly better speech perception in particular noise conditions. No effect of WNR on FMS in quiet or SRTs in noise was found in the absence of wind. Concerning speech perception in quiet and subjective auditory benefit, the performance was comparable between the DUET 2 and SONNETeas audio processors.

## Supporting information

S1 Table Table including the individual data of speech tests and questionnaires. (XLSX)
